# Regulation of correlative inhibition of axillary bud outgrowth by basal branches varies with growth stage in *Trifolium repens*

**DOI:** 10.1093/jxb/erv184

**Published:** 2015-04-28

**Authors:** Roderick G. Thomas, Michael J. M. Hay

**Affiliations:** AgResearch Grasslands, Private Bag 11008, Palmerston North, New Zealand.

**Keywords:** Apical dominance, auxin, branching regulation, correlative inhibition, cytokinin, prostrate clonal herbs, white clover (*Trifolium repens*).

## Abstract

In *Trifolium repens* the decline in bud outgrowth that occurs with distance from basal root systems is due to correlative inhibition by the first-formed basal branches. The apical and axillary buds on these basal branches are the source of the inhibitory effect, but their mode of action is unclear. Inhibition might occur via basal branches being a sink for xylem-transported branching stimulants or alternatively by providing a source of inhibitory signals, or by both mechanisms. To distinguish between these mechanisms, four experiments were conducted on plants varying in initial growth stage from 10 to 19 nodes along their main stems to determine any variation in the relative importance of the operative mechanisms of correlative inhibition. Inhibitory signal exported into the main stem, detected as a branching response to girdling of basal branches, was relatively more significant in smaller (initially with 10–15 nodes on the main stem) than in larger (>19 nodes on main stem) plants. This signal was shown not to involve auxin fluxes, and is unidentified. However, across all stages of growth, the predominant mechanism driving correlative inhibition was the action of axillary and apical buds of basal branches as sinks for the stimulatory signal. This study indicates that the relative importance of the mechanisms regulating bud outgrowth in *T. repens* varies with growth stage and that, during intermediate stages, regulation has some similarity to that in *Pisum*.

## Highlight

During growth, regulation of distal bud outgrowth by an inhibitory non-auxin signal becomes increasingly overshadowed by the negative influence of basal branch sink activity for a root-derived stimulatory signal.

## Introduction

The regulation of axillary bud outgrowth is a major determinant of the development of plant forms adapted to the environmental conditions in which they are growing (Leyser, 2009). The significance of such regulation has led to its intensive study in the model systems of *Arabidopsis*, *Pisum*, and *Petunia* and the discovery that it is influenced by a network of systemically moving endogenous signals (Ongaro and Leyser, 2008; Ferguson and Beveridge, 2009; Dun *et al.*, 2009; Drummond *et al.*, 2009; Domagalska and Leyser, 2011; Cheng *et al.*, 2013; Shinohara *et al.*, 2013). Ferguson and Beveridge (2009) showed that in *Pisum sativum* the inhibitory processes of apical dominance, correlative inhibition by basal branches, and the strigolactone hormone signalling pathway all had independent influences but in combination acted to regulate bud outgrowth. In the erect-stemmed species, *Arabidopsis* and *Pisum*, apical dominance is strongly expressed and while its mechanism of operation is currently debated (Brewer *et al.*, 2013; Shinohara *et al.*, 2013) the central active role of polar-transported auxin is widely acknowledged. The mode of action of correlative inhibition by basal branches is less clear but the export of auxin (Ongaro *et al.*, 2008) and feedback signals (Dun *et al.*, 2012) have been suggested as being important components. Once again, the mode of action of strigolactone is debated and thought to have its main effect either by directly repressing bud outgrowth (Gomez-Roldan *et al.*, 2008; Brewer *et al.*, 2009; Dun *et al.*, 2012, 2013) or by moderating auxin flux by reducing PIN1 accumulation on the plasma membrane of cells in the polar transport pathway (Crawford *et al.*, 2010; Liang *et al.*, 2010; Shinohara *et al.*, 2013). These inhibitory processes are counterbalanced by the hormone cytokinin, a branching stimulant, which is transported acropetally in the xylem and synthesized in both roots and stem tissues (Sachs and Thimann, 1967; Chen *et al.*, 1985; Nordström *et al.*, 2004; Tanaka *et al.*, 2006). Direct application of cytokinin promotes the outgrowth of buds (Cline, 1991). However, cytokinin activity is internally modulated as its biosynthesis and degradation are down- and upregulated, respectively, by auxin (Nordström *et al.*, 2004; Tanaka *et al.*, 2006; Werner *et al.*, 2006; Shimizu-Sato *et al.*, 2009) and there is a direct antagonistic effect of strigolactone on cytokinin acting through BRC1 (BRANCHED 1/TEOSINTE BRANCHED 1-LIKE) at the bud (Dun *et al.*, 2012). Recently sugar was also identified as a promoter of bud outgrowth in *Pisum*, its increased supply to buds following decapitation of the primary stem acting to stimulate bud outgrowth (Mason *et al.*, 2014). Thus there is a network of interacting shoot- and root-derived signals together with feedback mechanisms that operate as regulators of branching in the erect-stemmed model plant systems in which the inhibitory processes play an important role.

**Fig. 1.**
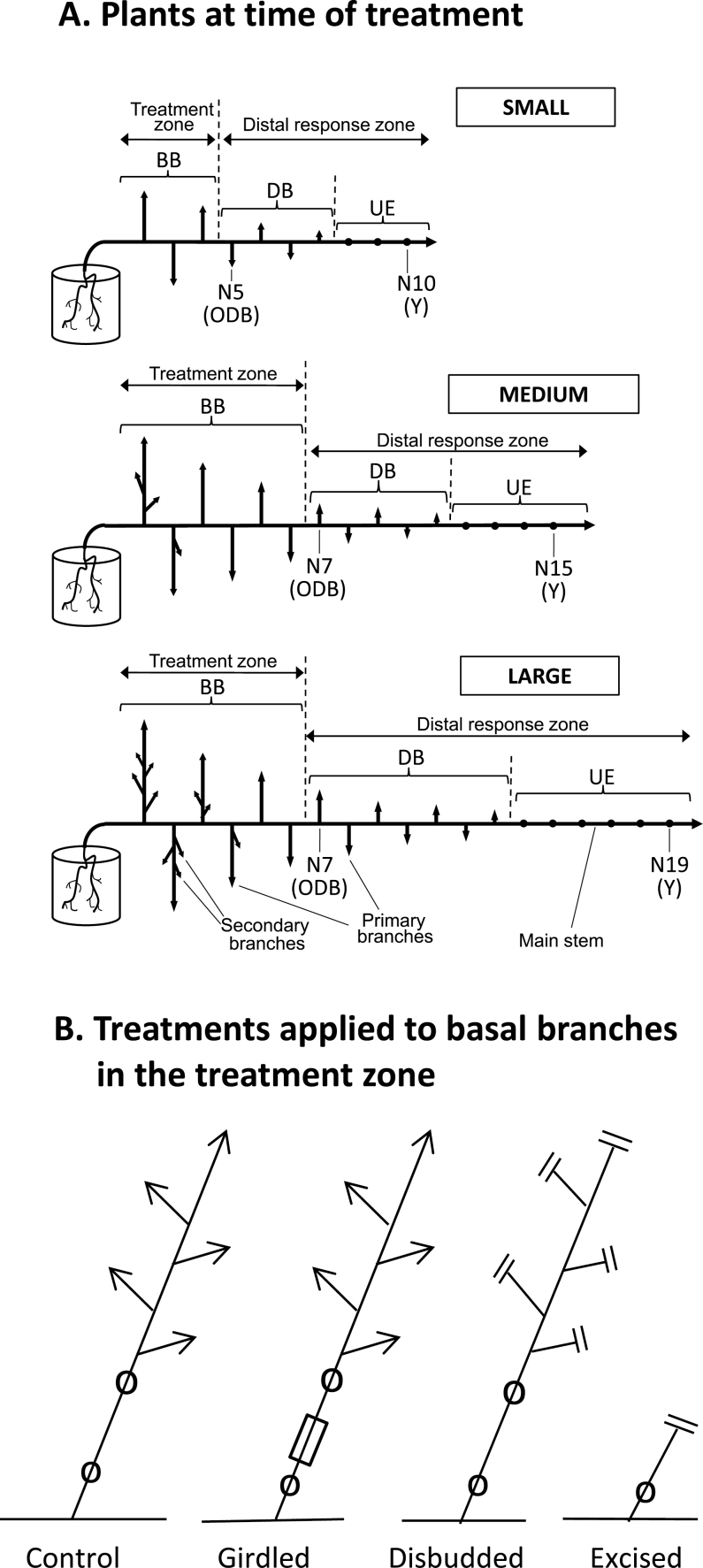
The core design and experimental procedures for Experiments 1–3: (A) growth stages of the plants at the time of treatment application for plants designated as small, medium, and large. DB and UE are distal branches and unemerged buds in the distal response zone, respectively; BB, basal branches in the treatment zone; ODB, the oldest branch in the distal response zone; Y indicates the position of the node (N) bearing the youngest fully unfolded leaf on the main stem. Nodes on the main stem are numbered acropetally from its base. (B) For each treatment, a diagram of one of the identically treated basal branches in the treatment zone for control, girdled, disbudded, and basal branch excision treatments. Open circles depict the nodes from which axillary buds and leaves were removed; arrows indicate intact active axillary or apical buds; and short double lines indicate points of stem excision or axillary bud removal. The open rectangle shows the position of the wax girdle.

In contrast to the situation in the erect-growing annual species, in the nodally rooting prostrate perennial clonal herb *Trifolium repens* the regulatory effect of apical dominance on axillary bud outgrowth into branches is relatively minor (Thomas *et al.*, 2003b) and the primary control of axillary bud outgrowth is via a net root-derived stimulus (NRS) (Thomas and Hay, 2007, 2008, 2014; Thomas *et al.*, 2014). As a horizontal main stem grows away from its basal root systems without further nodal root formation, its six to eight basal-most axillary buds grow out vigorously into branches (Fig. 1A: BB). Branching vigour of successively later-formed distal axillary buds (Fig. 1A: DB) then declines markedly until the latest to form remain within the stipular sheaths surrounding them (Fig. 1A: UE) (Thomas *et al.*, 2002). Following this decline, outgrowth of an otherwise unemerged bud at a distal node can be triggered by the formation of a nodal root at that node (Lötscher and Nösberger, 1996; Thomas *et al.*, 2002; Thomas and Hay, 2007, 2015). Indirect evidence suggests the likelihood that the NRS is xylem-transported cytokinin (Thomas and Hay, 2015).

The decline in branching vigour along the main stem is the result of correlative inhibition by the first-formed basal branches, as evidenced by the stimulatory effect of their excision on distal branching (Thomas *et al.*, 2003a, b; Thomas and Hay, 2014). Excision of all buds from these branches while their stems and foliage remain intact has a comparable effect (Thomas *et al.*, 2003a), demonstrating that buds are a major inhibitory influence. Whether this inhibitory effect is brought about by the action of these buds as sinks for NRS or by the export of inhibitory compound(s) from them, or by both mechanisms, is unclear. A previous study, using large highly branched plants (Thomas and Hay, 2011), strongly supported the overriding importance of the sink activity of basal branches; it provided no evidence for export of inhibitory signals from them. This evidence obtained for *T. repens* contrasts with that from experiments with *Pisum* (Beveridge *et al.*, 1996; Dun *et al.*, 2009; Ferguson and Beveridge, 2009), which have led to the suggestion that, in that species, branches may play a role in the regulation of bud outgrowth via the export of an inhibitory signal.

These contrasting results for *T. repens* and *Pisum* might result from changes that occur in *T. repens* during shoot growth away from its basal root system, as evidenced by changes in the relative importance of apical dominance. In addition to the central role of NRS in the regulation of axillary bud outgrowth, apical dominance has a significant regulatory influence at some growth stages (Thomas *et al.*, 2003b). Initially, up to the time when the sixth node distal to the basal root system has emerged, the stimulatory strength of NRS supplied by the basal root system is high and the apical bud on the main stem has no inhibitory influence as judged by lack of response to its excision. This initial phase is followed, as further nodes emerge on the main stem and sink strength of the increasingly large basal branches for NRS increases, by one in which the concentration of NRS appears gradually to decline leading to a decrease in outgrowth of later-formed buds. During this second phase, in which nodes 8 to 15 emerge on the main stem and plants attain a medium size (see Fig. 1A), outgrowth of the most recently emerged axillary bud is stimulated by excision of the apical bud distal to it. This is followed by a third phase, from emergence of node 16 onwards, when the availability of NRS systemically throughout large plants (Fig. 1A) appears limiting for axillary bud outgrowth (Thomas and Hay, 2008) and, as a result, apical bud excision has no stimulatory effect (Thomas *et al.*, 2003b).

The second phase, during which experimental evidence (Thomas *et al.*, 2003b) suggests NRS supply to newly emerging axillary buds is inadequate to stimulate their outgrowth in the presence of their apical bud, but allows their outgrowth following apical bud excision, appears comparable to the situation prevailing in the well studied erect-growing species.

The present study was therefore undertaken primarily to test the hypothesis (Hypothesis 1) that in smaller, less highly branched, plants of *T. repens* basal branches act similarly to those in *Pisum* by exporting an inhibitory signal into the main stem. Hot wax girdling of basal branches, designed to block such export via the symplast, gave results supporting this hypothesis. A second hypothesis (Hypothesis 2) that in smaller plants the exported signal might be auxin related as proposed for *Arabidopsis* (Ongaro *et al.*, 2008) was not supported by the tests imposed, and neither was a third (Hypothesis 3) that, in the absence of sink activity by branch buds following their excision, a stimulatory signal (NRS or a derivative) is re-exported into the main stem. The possibility (Hypothesis 4), that in larger plants the failure of hot-wax girdling of basal branches to stimulate distal bud outgrowth (Thomas and Hay, 2011) resulted from the inhibitory effect of well developed branches present in the older regions of the distal response zone (Fig. 1A: DB) was supported by the experimental results.

## Materials and methods

### Plant materials

*Trifolium repens* L. (white clover) plants used were derived from a greenhouse-grown stock clone of a single genotype selected from a Spanish ecotype collection (AgResearch Accession number C1067) as previously described (Thomas *et al.*, 2002; Thomas and Hay, 2007, 2008).

### Definition

Throughout this study, branches were deemed to include all actively growing buds, as evidenced by the occurrence of leaf emergence on them, in addition to axillary shoots with elongated stems.

### Hormone materials

Hydrous lanolin paste, used in control treatments, was produced by adding water to melted anhydrous lanolin in the ratio 3:2 (lanolin:water) by weight and then vigorously stirring the mixture.

For 1-naphthaleneacetic acid (NAA) treatments, NAA dissolved in a drop of ethanol was added to the hydrous lanolin paste (10mg of NAA g^–1^ hydrated lanolin) and mixed thoroughly.

For *N*-1-naphthylphthalamic acid (NPA) treatments, NPA dissolved in ethanol was added to the hydrous lanolin paste (10mg of NPA g^–1^ hydrated lanolin) and mixed thoroughly.

All lanolin-based treatments were applied twice weekly.

### Culture of experimental plants

Plants were grown from stem tip cuttings planted in a commercially obtained potting mix (Thomas *et al.*, 2002) in 1.8 l plastic pots. After 3 weeks, the two or three basal-most branches formed were trimmed off each plant to leave a single main stem axis growing away from its basal root system. All lateral branches that grew out subsequently from this main stem were retained (Fig. 1A). The oldest node on the main stem that retained a branch was termed node 1 (N1) and successively later-formed ones termed N2, N3, etc. Outgrowth of nodal roots was prevented by growing shoot systems out over a dry plastic mesh. Throughout all experiments, plants were grown in a heated greenhouse in natural photoperiods at average maximum/minimum temperatures of 25/12°C.

## Experimental methods

There were three experiments in which the basic design was similar, but plants differed in growth stage. Each plant was made up of two zones: a basal treatment zone bearing the oldest retained branches (Fig. 1A: BB) and a distal response zone in which the axillary buds at nodes along the main stem had either grown out into short branches (Fig. 1A: DB) at the time of treatment or remained unemerged from the stipular sheaths at the bases of their subtending leaves (Fig. 1A: UE).

The growth stage attained by each plant at the time experimental treatments were imposed was assessed by measuring the lengths of all branches and main stems and counting the number of emerged leaves (or nodes) on each using the Carlson scale of leaf development (Carlson, 1966). The branch sizes at this time, expressed as total number of nodes present on them in the basal (BB) and distal (DB and UE) zones are shown in Table 1.

**Table 1.** Total number of emerged nodes on basal and distal branches at the time treatments were applied to different-sized plants in Experiments 1–3

Experiment	Plant size	BB	DB
1	S	20	9
	M	76	12
	L	179	29
2	S	38	14
3A and 3B	M	84	19

BB, basal branches; DB, distal branches; S, small plants; M, medium plants; L, large plants.

Treatments were applied to the basal branches and stem lengths of, and numbers of leaves on, axillary buds and branches reassessed after periods ranging from 7 to 19 days. In all experiments, plants were grown until the basal branches to be treated had each formed ≥ four expanded leaves. Axillary buds and their subtending leaves were then excised from the basal-most two nodes on each, after which three, or all of the following four, basic treatments were imposed (Fig. 1B):

(i) Control: basal branches left untreated.
(ii) Girdled: basal branches girdled midway along their second oldest internode. Girdling was imposed by applying molten wax, heated to 110°C, via a custom-built Perspex chamber, to a 10mm segment of this internode. This killed all live cells across the stem segment as described by Thomas and Hay (2011).
(iii) Disbudded: basal branches disbudded by excising all their axillary and apical buds, leaving their stems intact and fully foliated.
(iv) Excised: basal branches excised distal to their lower-most, oldest node by cutting through the midpoint of their second oldest internode and leaving the base of the branch as a stump.

### Experiment 1

This was designed to test Hypothesis 1 that the branching response to girdling of basal branches varies with plant growth stage.

Treatments were applied to plants of three growth stages [small (S), medium (M), and large (L)] that related in particular to the extent of development of their basal branches. Preparation of material was staggered so that plants of different sizes could receive their treatments simultaneously. Thus, 24 cuttings for large plants were taken from stock plants on 21 February, 24 for medium plants on 7 March, and 24 for small plants on 21 March (all 2011). Treatments were applied to each of the basal branches from 4–6 May, when the youngest fully expanded leaf (Y) on each main stem was situated at node 19 (N19) on large plants, N15 on medium plants, and N10 on small plants (Fig. 1). In each size group three basic treatments (control, girdled, or disbudded) were imposed with each replicated eight times and bud outgrowth responses in the distal response zone assessed 7 days after the start of treatments.

### Experiment 2

Treatments were applied to small plants to test Hypothesis 2 that, in contrast to the situation in larger plants (Thomas and Hay, 2011), the suppression of distal branching by basal branches results from their export of auxin into the main stem.

Fifty cuttings were taken on 15 June 2011 and grown on until 16 August when 30 uniform plants were selected for the experiment. At this time plants were slightly larger than the small plants of Experiment 1 (Fig. 1A), with four basal branches in the treatment zone instead of three and the oldest branch in the distal response zone at node 5. The youngest fully expanded leaf (Y) on the main stem was at node 12. Total branch sizes at this time are shown in Table 1.

The five treatments imposed, replicated six times, consisted of three basic ones (control, girdled, and excised), together with two additional hormone treatments similar to those described in Thomas and Hay (2011) as follows:

(i) NPA: intact plants as in control plants but with NPA in hydrous lanolin paste applied around the midpoint of the second oldest internode of each of the four basal branches.
(ii) NAA: basal branches removed as in the excised treatment (Fig. 1B), but NAA in hydrous lanolin paste applied to the remaining stumps immediately after branch excision.

Lanolin paste was applied to the control and excised treatments as appropriate for controls, respectively, for the NPA and NAA treatments.

Outgrowth responses of axillary buds in the distal response zone (buds at node 5 and onwards) were assessed 14 days after treatments were applied.

### Experiment 3

This experiment consisted of two parts, A and B. Ninety cuttings were taken on 31 August 2010 and grown on until 8 October, when 40 uniform plants were selected for Experiment 3A and 24 for 3B. At this time there were six basal branches in the treatment zone and the youngest unfolded leaf on each plant was situated at the 15th node of the main stem, as shown for the ‘Medium’ plant in Fig. 1A. Branch sizes at the time of treatment are shown in Table 1.

### Experiment 3A

This was designed to test Hypothesis 3 that the known stimulatory effect of disbudding basal branches (Thomas *et al.*, 2003a) results from their consequent export of a stimulatory signal into the main stem via the symplast. Should this hypothesis be incorrect, the prediction would be that girdling of the disbudded branches would have no influence on their stimulatory effect.

The five treatments imposed, replicated eight times, consisted of the four basic treatments (control, girdled, excised, and disbudded) together with an additional treatment (disbudding plus girdling) in which the six basal branches were disbudded as in the disbudding treatment and then hot-wax girdled 10 minutes later, as in the girdled treatment.

Outgrowth of axillary buds in the distal response zone was recorded after 14 days. For plants in the control and girdled treatments only, all axillary buds on the oldest untreated distal branch, at node 7 on the main stem (Fig. 1A: ODB), including the oldest three unemerged buds within the apical bud of that branch, were measured under a dissecting microscope at this time.

### Experiment 3B

This was designed to test Hypothesis 4 that the presence of well developed distal branches in the response zone (Fig. 1A: DB) might play an inhibitory role over and above that played by the basal branches in the treatment zone.

Four treatments (control, girdled, excised, and disbudded), with six replicates of each, were set up with all distal branches (DB) and unemerged buds (UE) excised from node 7, immediately adjacent to the basal treated zone, through to and including node 14. Bud outgrowth responses were then measured in the apical region from node 15 onwards after 9, 14, and 19 days for direct comparison with those in Experiment 3A.

### Dry weights

At the end of Experiments 2 and 3A, plants were divided into four portions: the branches in the basal treatment zone; all branches and axillary buds in the distal response zone; the whole of the main stem including its leaves; and the basal root system. Dry weights of these portions were then determined after drying to constant weight for 4 days in a draught oven at 60°C.

### Data analysis

In all experiments, for each of the assessed characteristics, data were tested for homogeneity of variance and analysed by ANOVA in the GENSTAT (16th edition) statistical package (VSN International, Hemel Hempstead, UK). Means and differences between them were conservatively evaluated using the relevant LSD_5%_ value obtained from the appropriate interaction table.

## Results

### The influence of plant growth stage

Disbudding of the basal branches in the treatment zone tended to increase the length of the main stem across all three plant size classes during the 7-day period of Experiment 1 (Supplementary Table S1) with the increases reaching significance in medium- and large-sized plants. Increases in numbers of emerged leaves were much more muted and only significant for medium-sized plants. Girdling of the basal branches significantly reduced main stem elongation (from 64.6 to 52.6mm) in large plants but did not affect the two groups of smaller-sized plants.

**Fig. 2.**
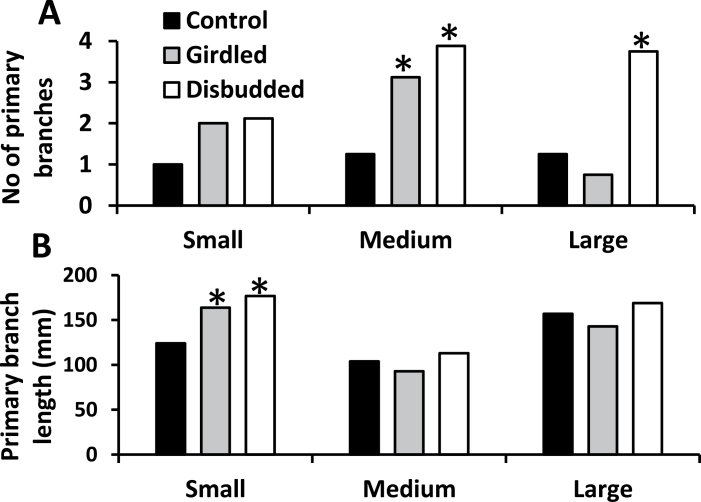
For Experiment 1, the increase in (A) the number of primary branches and (B) their total length in the distal response zone during the 7-day period following treatment application for the control, girdled, and disbudded treatments in small-, medium-, and large-sized plants, n = 6. Asterisks indicate significantly (*P* < 0.05) different values from the control treatment.

Treatment responses of axillary buds in the distal response zone differed among the three size classes. For increase in number of buds developing into primary branches in small plants there was a non-significant doubling of values in response to girdling or disbudding of basal branches relative to the control whereas increases were significant in medium-sized plants and, in response to basal branch disbudding only, in large plants (Fig. 2A). Increases in primary branch stem length in response to both girdling and disbudding were significant in small plants whereas medium- and large-sized plants showed no significant response (Fig. 2B).

### Localization of branching in response to girdling

**Fig. 3.**
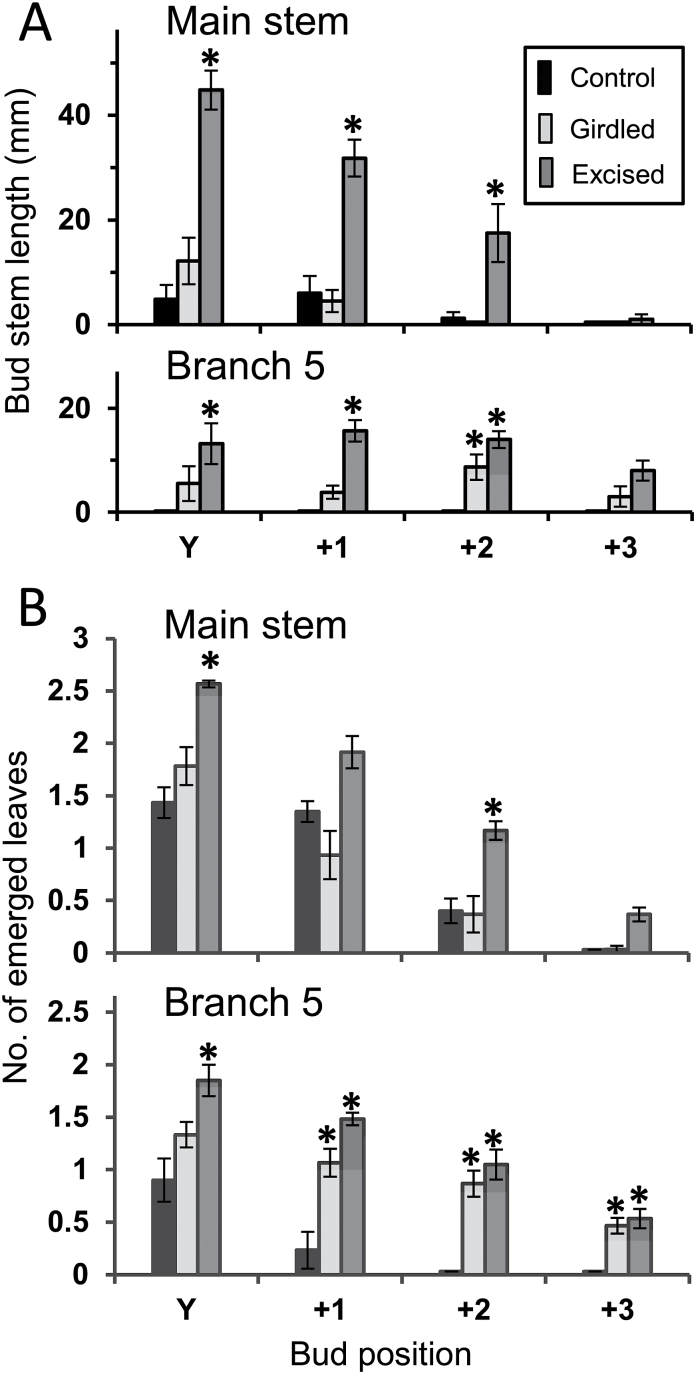
Growth of axillary buds at nodes emerged successively from the apical buds of the main stem and branch 5 (the oldest branch in the distal response zone) during the 14-day period following treatment application in Experiment 2. Size of buds at the end of that period was measured as (A) the length of their stems and (B) the number of leaves that had emerged on them. Y is the node bearing the youngest unfolded leaf at the start of the experiment; +1, +2, and +3 are successively later-emerged nodes. Plants either remained intact (control) or their basal branches were girdled or totally excised; *n* = 6. Error bars indicate SE of means and asterisks indicate values significantly (*P* < 0.05) different from the control value.

**Fig. 4.**
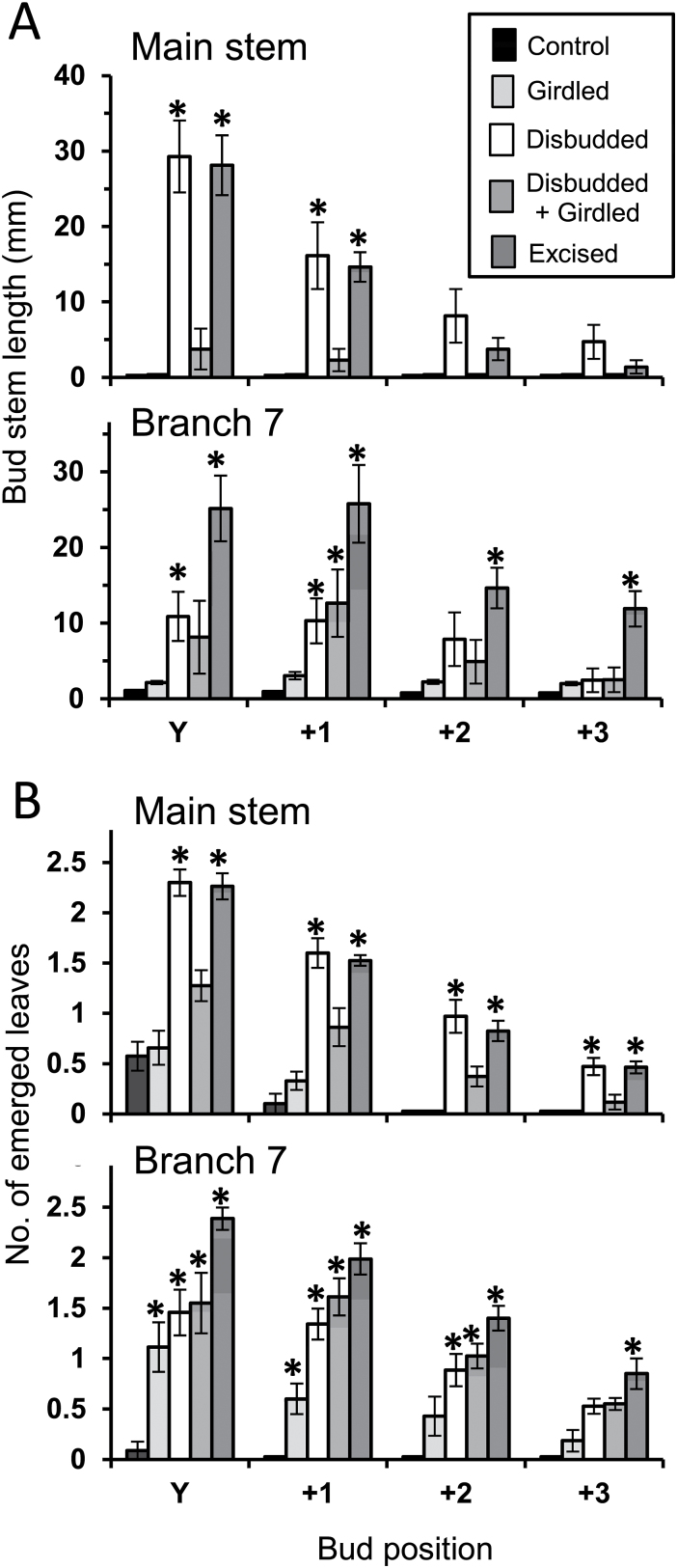
Growth of axillary buds at nodes that emerged successively from the apical buds of the main stem and branch 7 (the oldest branch in the distal response zone) during the 14-day period following treatment application in Experiment 3A. Bud sizes at the end of that period are presented as (A) the lengths of their stems and (B) the number of leaves that had emerged on them. Y is the node bearing the youngest unfolded leaf at the start of the experiment; +1, +2, and +3 are successively later-emerged nodes. Plants either remained intact (control) or their basal six branches were girdled, disbudded, disbudded and girdled, or totally excised; *n* = 8. Error bars indicate SE of means and asterisks indicate values significantly (*P* < 0.05) different from the control value.

In the smaller- and medium-sized plants in Experiments 2 and 3, while the stimulation of branch outgrowth in response to basal branch excision was very strong, both at the growing tip of the main stem and at the tip of the oldest branches in the distal response zone (Fig. 1A: ODB), the responses to girdling differed in these two regions (Figs 3 and 4). In both experiments girdling failed to stimulate axillary bud outgrowth at nodes that emerged from the apical bud of the main stem during the 14-day response periods. Girdling did, however, stimulate branch outgrowth at nodes that emerged from the apical bud of the oldest branch in the distal response zone (branch 5 in the smaller plants in Experiment 2 and branch 7 in the medium-sized plants in Experiment 3A).

**Fig. 5.**
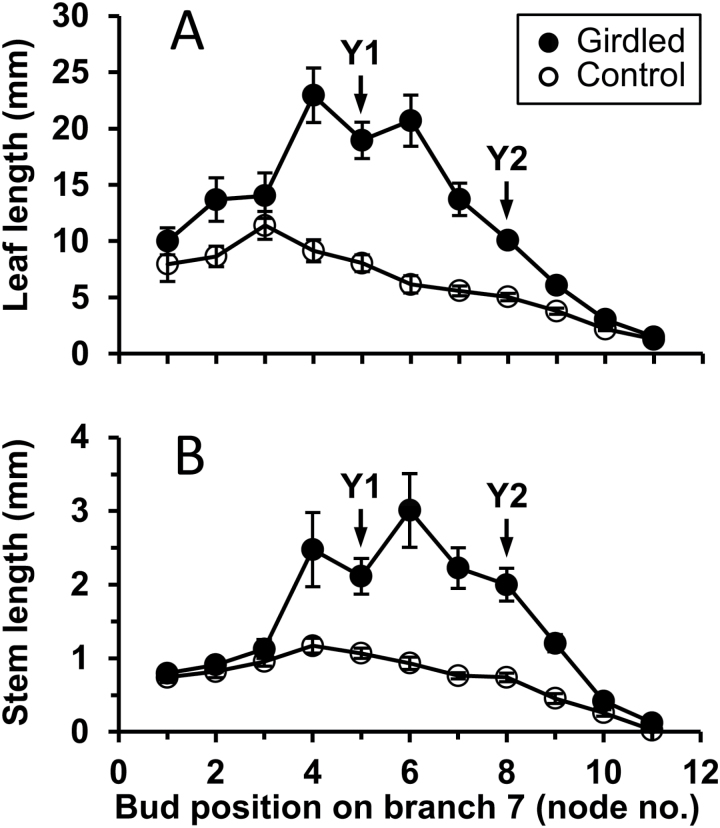
Axillary bud growth on branch 7 (the oldest intact branch in the distal response zone) in response to girdling of basal branches in Experiment 3A. Basal branches on control plants remained intact. Measurements of the length of each bud’s second oldest leaf (A) and stem (B) were made with the aid of microscopic examination at the end of the 14-day response period. Node 1 was the oldest node at the base of the branch and nodes 2, 3, etc. were successively younger. At the time of treatment, node 5 bore the youngest unfolded leaf (Y1); after 14 days the youngest unfolded leaf (Y2) was at node 8 on the branch and the axillary buds at nodes 9 to 12 were still within the branch’s apical bud; *n* = 8. Error bars indicate SE of means.

The sensitivity of buds on the oldest distal branch (branch 7) in Experiment 3A varied with their position on the branch (Fig. 5). Of the buds in axils of fully opened leaves at the time of treatment (at nodes 1 to 5), only the youngest two responded to basal branch girdling. At the end of the 14-day treatment period, the oldest buds, at nodes 1 to 3, remained small and not significantly different in size from those in control plants. The two younger buds, at nodes 4 and 5, and those in the axils of the leaves that emerged from their parent apical bud during the experiment (at nodes 6 to 8 on branch 7) were all up to three times larger in the girdled plants. This stimulatory influence of girdling clearly acted upon buds before their subtending leaves were fully expanded, as shown by the response of the bud at node 9 (Fig. 5).

In neither Experiment 2 nor 3A was the boost to bud outgrowth as great in response to girdling as to basal branch excision, with the difference between these responses being much greater in the larger plants in Experiment 3A. In addition, the relative stimulation in response to girdling decreased from node to node at successively formed nodes from Y to +3 on branch 7 (Fig. 4B).

### Branching response to distal branch excision

**Fig. 6.**
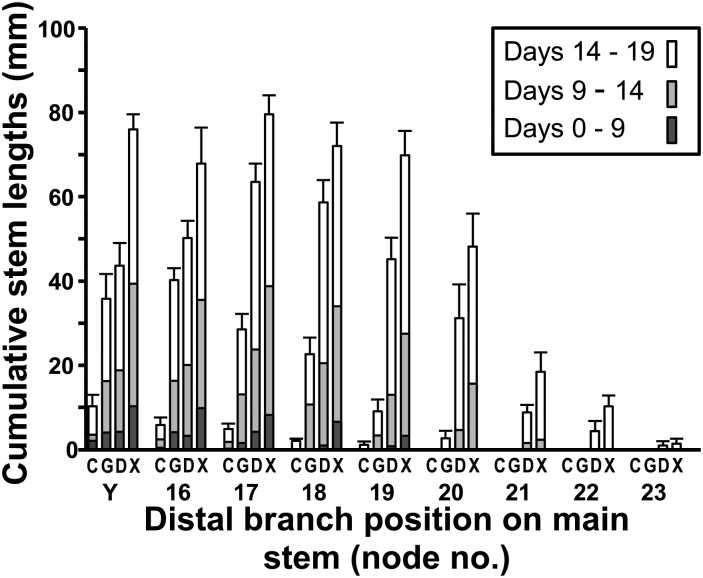
Axillary bud stem lengths at newly emerged distal nodes on the main stem in response to treatment in Experiment 3B. Basal branches either remained untreated (C) or were girdled (G), disbudded (D), or totally excised (X); *n* = 6. At the time of treatment application the youngest unfolded leaf (Y) was at node 15 on the main stem. Nodes that emerged successively from the main stem apical bud over a 19-day period are numbered 16–23. Error bars indicate SE of means.

Failure of basal branch girdling to stimulate branch outgrowth in the apical region of the main stem (Figs 3 and 4) was overcome by excision of all the axillary buds and young branches from the distal response zone in Experiment 3B immediately prior to treatment of the basal branches. This is clearly seen by comparison of bud outgrowth at the main stem tip in response to girdling in Experiment 3A (Fig. 4) with that in Experiment 3B (Fig. 6). In contrast to the lack of stimulation in Experiment 3A, in which all buds and branches in the distal response zone were retained, strong bud outgrowth occurred in response to girdling in Experiment 3B in the absence of branches in the distal response zone. Even in this case, however, the initial 9-day response to total excision of basal branches was two to three times greater than that brought about by girdling and the positive response to the latter diminished over time from node to node.

### Distal branching in response to disbudding of basal branches

Disbudding of basal branches in Experiments 3A and 3B strongly stimulated bud outgrowth in the apical region of the main stem. Stimulation in Experiment 3A (Fig. 4) was equal to that induced by total excision of basal branches, but significantly less in the apical region of branch 7. In the absence of branches in the distal response zone in Experiment 3B, the stimulatory response at the tip of the main stem was delayed after disbudding relative to that induced by branch excision (Fig. 6). Although the outgrowing branches were longer in the latter case, the elongation rates in response to these two treatments ultimately became the same, particularly at nodes 16 to 20, over the final 5 days of the experiment (days 14–19).

## The interaction between girdling and disbudding

In the absence of branches in the distal response zone in Experiment 3B the stimulatory effects of disbudding and girdling were initially similar (Fig. 6). From the third node (at node 17) onwards that emerged from the main stem’s apical bud after treatments were applied, however, the stimulatory responses to girdling fell markedly behind. When the branches in the distal response zone were retained in Experiment 3A, though, the marked stimulatory influence of disbudding contrasted strongly with the lack of branch outgrowth in response to girdling (Fig. 4).

The interaction between girdling and disbudding in Experiment 3A differed between the apical region of the main stem and that of branch 7 in a similar way to that described above for the difference in response to girdling alone. At the tip of the main stem, not only was there no stimulatory response to girdling, but girdling of disbudded basal branches reduced their stimulation of leaf emergence on buds by 50% (Fig. 4A) and stem length of outgrowing buds by even more (Fig. 4B). On branch 7, however, on which the stimulatory effect of disbudding was approximately halved in comparison with that of total branch excision, girdling of disbudded basal branches did not reduce this response.

### Branching in response to hormone treatments

**Fig. 7.**
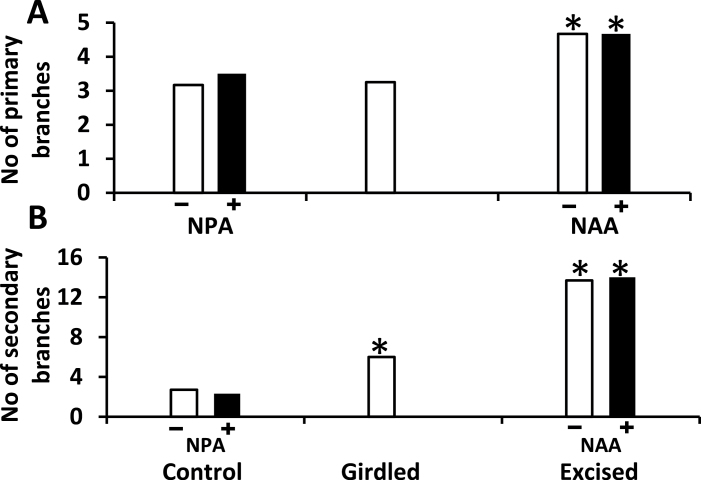
For Experiment 2, the increase in number of primary (A) and secondary (B) branches in the distal response zone over the 14-day period following treatment application; *n* = 6. Treatments: application (+) or not (–) of NPA to basal branches of intact plants; application (+) or not (–) of NAA to stumps of excised basal branches; or girdling of each of the intact basal branches. Asterisks indicate significantly (*P* < 0.05) different values from the control treatment.

Appropriate treatment of basal branches, or their post-excision stumps, with NPA or NAA had no effect on bud outgrowth in the distal response zone of small plants in Experiment 2 (Fig. 7) or, in the case of NPA treatment, on branch outgrowth on the basal branches themselves (Supplementary Table S2). Growth at the apex of the main stem, and total numbers of primary and secondary branches, were all completely unaffected by hormone treatments of the small plants (Fig. 7). Supplementary trials on spare plants showed that the NAA and NPA hormone treatments were effective in altering auxin transport within *T. repens* as both rapidly induced bending when applied to one side of a petiole.

### Response of treated basal branches to girdling

The impact of girdling on the growth and branching of basal branches in the treatment zone over the 14-day period of Experiment 2 was slight (Supplementary Table S2). Of the four branching characteristics observed, only the reduction in total length of secondary branches of girdled plants marginally reached significance at the 5% level of probability.

Application of girdles to basal branches had the aim of preventing export of inhibitory influences from them via the symplast while allowing continued passage of root-derived products into them via the xylem. This aim was largely achieved: girdled branches remained fully turgid throughout, indicating that there was no restriction of water flow into them through the xylem; their sink activity was little impaired as indicated by a reduction in their growth by only ~10%; and girdles did not prevent outgrowth of their axillary buds. In addition, the reduction in growth of the basal root systems in response to girdling in both intact and disbudded plants (Table 2) to the same level as for plants totally lacking basal branches suggests that girdles successfully prevented the export of photosynthate via the symplast.

**Table 2.** Dry weights (grams) of plant components 14 days after treatment application in Experiments 2 (*n* = 6) and 3A (*n* = 8) for treatments in which basal branches either remained intact, or were girdled, disbudded, disbudded and girdled, or totally excised

	Treatments in which basal branches either remained intact (C), or were girdled (G), disbudded (D), disbudded and girdled (D+G), or totally excised (X)				
	C	G	D	D + G	X
Experiment 2:					
Main stem	1.1^a^	1.0^b^	–	–	1.2^a^
Distal branches	1.1^a^	1.3^a^	–	–	1.8^b^
Basal branches	4.9^a^	5.3^a^	–	–	–
Experiment 3A:					
Main stem	1.2^b^	1.1^a^	1.5^c^	1.3^b^	1.4^c^
Distal branches	1.9^a^	1.9^a^	3.5^c^	2.7^b^	3.5^c^
Basal branches	11.4^a^	12.0^a^	–	–	–
Basal root	1.6^b^	0.9^a^	1.9^c^	0.9^a^	0.9^a^

Within rows, means without a common superscript differ (*P* < 0.05).

### Dry weights

In both the smaller plants used in Experiment 2 and the medium-sized ones in Experiment 3A, final dry weights of component shoot parts were little influenced by girdling of basal branches over the 14-day duration of the experiments, although the overall dry weights of the main stems were slightly but significantly reduced (Table 2). This contrasted with the stimulatory effects of disbudding and total excision of basal branches on the dry weight gain of main stems and distal branches.

In Experiment 3A, final basal root biomass was higher following disbudding of basal branches than in control plants (1.9 vs 1.6g), but very much lower (0.9g) following girdling, girdling plus disbudding, or basal branch excision.

## Discussion

### Basal branches export an inhibitory signal (Hypothesis 1)

The lack of evidence for the export of inhibitory signals from the basal branches of large highly branched plants of *T. repens* has led to the suggestion that these branches act largely as sinks for NRS (Thomas and Hay, 2011). This is contrary to the case in the less highly branched orthotropic species *Pisum* (Beveridge *et al.*, 1996; Ferguson and Beveridge, 2009; Dun *et al.*, 2009) and *Arabidopsis* (Ongaro *et al.*, 2008), in which the role of basal branches in correlative inhibition has been attributed to their export of inhibitory signals. A primary aim of the present study was to test Hypothesis 1 that in smaller, less highly branched plants of *T. repens* the basally located branches restrict outgrowth of distally located axillary buds, at least to some extent, via the export of an inhibitory signal. To test this possibility, hot-wax girdles were applied near the bases of the basal branches to prevent export of inhibitory influences via the symplast while allowing root-derived products into these branches via the xylem. The results of Experiment 1 showed that this hypothesis was correct. They confirmed the previous observation (Thomas and Hay, 2011) that there was no measurable stimulatory response to girdling in large plants (Fig. 2) but showed clearly that it stimulated axillary bud outgrowth in the distal response zones in small- and medium-sized plants.

### The inhibitory influence of distal branches (Hypothesis 4)

From a more detailed study of this stimulatory response in smaller plants (Experiments 2 and 3A), it is clear that stimulation was confined to the oldest branches in the distal response zone, not being detectable in the younger, more distal region of the main stem (Figs 3, 4 and 7). Figs 3 and 4 show the contrast between the response of axillary buds that newly emerged from their parent apical bud on the oldest untreated branch in the distal response zone and of those that emerged from the main stem apical bud during the 14 days following girdling. These differences are possibly related to differences in branch development immediately proximal to the apical buds at the time of treatment. At the time of girdling, the oldest distal (primary) branches (Fig. 1A: ODB) bore no secondary branches between their apical bud and their point of attachment to the main stem immediately distal to the basal treatment zone. In the case of the apical bud on the main stem, however, several well developed branches in the distal response zone were present between it and the basal treatment zone. The probable inhibitory role of such actively growing branches is seen by comparing the results of Experiments 3A and 3B (Figs 4 and 5), designed to test Hypothesis 4 that their presence causes the lack of response to girdling in the apical region of the main stem in the intact medium-sized plants. In contrast to the lack of stimulation of bud outgrowth seen in fully branched plants in Experiment 3A, there was a marked stimulation of bud outgrowth in response to girdling in plants in Experiment 3B from which all branches and buds in the distal response zone of the main stem (Fig. 1A) were excised immediately before the time of girdling. In the larger plants, as used in the previously reported study (Thomas and Hay, 2011) and in Experiment 1, the oldest branches in the distal response zone themselves bore secondary branches that would have similarly acted to prevent stimulation of distal bud outgrowth in response to girdling.

### The short-lived response to girdling

In Experiments 2, 3A, and 3B, the effect of girdling of the basal branches was relatively small after 14 days compared with that of total basal branch excision (Figs 3, 4 and 6). On the oldest distal branch (Fig. 5: branch 7) girdling strongly stimulated outgrowth only of newly emerged buds, having little or no effect on those older buds that were already emerged at the time treatments were applied. Total excision of basal branches stimulated outgrowth of all buds on these branches. Similarly, while the initial response to girdling was quite strong in buds at the first nodes to emerge after treatment, the response was only short lived, decreasing markedly from node to node in later-emerging buds. This showed particularly clearly in Experiment 3B (Fig. 6) in which the initial rate of bud outgrowth at the first two nodes that emerged (at Y and N16) did not differ from that in plants in which basal branches were disbudded but then declined rapidly at successively formed nodes. In contrast, growth of buds at the later-formed nodes was unrestricted following both disbudding and total excision of basal branches. Thus, while the stimulatory effect of girdling has provided evidence of the export of an inhibitory signal by actively growing branches, most of the effect of the presence of basal branches on the decline of branch outgrowth along the main stem must result from their possible role as sinks for NRS.

### Basal branches do not export a branching stimulus (Hypothesis 3)

Previous studies have shown that the inhibitory role of basal branches is attributable to their actively growing apical and axillary buds (Thomas *et al.*, 2003a; Thomas and Hay, 2011). Disbudding of basal branches leads to a similar, though somewhat delayed (Fig. 6), stimulation of bud activation to that caused by total branch excision (Thomas *et al.*, 2003a). Girdling of disbudded branches to test Hypothesis 3 that in addition to their export of an inhibitory signal, branches export a branching stimulant, suggested once again that the main effect of disbudding basal branches was caused by a decrease in their sink activity. In Experiment 3A, even though girdling alone stimulated bud outgrowth in the apical region of the oldest distal branch (Fig. 4: branch 7), there was an additional stimulatory effect of the disbudding plus girdling treatment over and above that of girdling alone on both the main stem and branch 7. In both girdling treatments export of compounds via the symplast would have been equally blocked, but disbudding probably reduced the loss of branching stimulus into the basal branches via their sink activity.

### The nature of the inhibitory signal from basal branches (Hypothesis 2)

The nature of the inhibitory signal exported from basal branches in *T. repens* is yet to be elucidated. Previously, when using large plants, Thomas and Hay (2011) found no evidence for a possible role of auxin as an inhibitory signal. The results of Experiment 2, designed to test Hypothesis 2 that auxin plays an inhibitory role in smaller plants, similarly failed to provide evidence to support the hypothesis; results of treatment with NAA as a substitute for the presence of basal branches and NPA to block auxin export showed remarkable similarity to untreated controls (Fig. 7). This lack of response to treatments manipulating the flow of auxin from basal branches into the main stem is important as it implies the stimulatory responses in distal tissues to girdling in small/medium-sized plants are not the result of variations in auxin flow influencing the regulation of the biosynthetic genes for cytokinin and strigolactone in the main stem (Nordström *et al.*, 2004; Foo *et al.*, 2005; Tanaka *et al.*, 2006; Hayward *et al.*, 2009). Indeed, Thomas and Hay (2014) showed in phosphorus-limited plants, where strigolactone biosynthesis is usually enhanced (Umehara *et al.*, 2010; Kohlen *et al.*, 2011; Brewer *et al.*, 2013), that responses to basal branch excision were not consistent with strigolactone having a significant role in the correlative inhibition imposed by basal branches. However, there remains the possibility that the prevention of flow of other compounds that act as inhibitors from branches into the main stem is responsible for the stimulatory effect of girdling in smaller-sized plants. The girdle would have blocked the transport of sucrose out of basal branches which mitigates against the possibility that the positive response to girdling resulted from increases in sucrose supply to buds (Mason *et al.*, 2014). Such a reduction in export of sugars to the basal root system could be expected to have reduced the roots’ metabolic activity and, as a result, led to a reduction in their production of NRS and in its export to the shoot. This in turn might explain the reduction in stimulation of distal axillary bud outgrowth in response to disbudding that was brought about by girdling disbudded basal branches.

### Do basal branches export a non-auxin inhibitory signal?

Overall, these results indicate that in *T. repens* regulation of bud outgrowth is dominated by the correlative inhibition imposed by previously formed basal branches. Across the range of growth stages examined, the correlative inhibition imposed by basal branches appears to be predominantly via the mechanism of their action as sinks for NRS. However, in the small- to medium-sized plants, plant sizes previously shown to express apical dominance (Thomas *et al.*, 2003b), there was a positive effect of girdling of basal branches on distal bud outgrowth (Figs 2, 5 and 6). This response was shown not to be mediated via the girdles preventing auxin flow from basal branches into the main stem (Fig. 7) and suggests the possibility that girdling prevented the export of a non-auxin inhibitory signal into the main stem as has been shown to occur in *Pisum* (Ferguson and Beveridge, 2009; Dun *et al.*, 2009).

### How widespread are these mechanisms of correlative control?

As detailed in the Introduction, three clear phases are discernible during growth of the shoot system from a basally rooted cutting in the absence of further nodal root formation (Thomas *et al.*, 2003b). These three phases seen in *T. repens* are common to many prostrate nodally rooting herbs (Thomas and Hay, 2004, 2010) and distinguish such species from the erect-growing annuals such as *Pisum*. The small- to medium-sized plants in this study, corresponding to plants in the second phase, show maximal responses to the export of an inhibitor from basal branches and positive bud outgrowth responses to decapitation (Thomas *et al.*, 2003b). Thus during growth through this phase *T. repens* plants show some similarity to *Pisum* in their regulation of bud outgrowth, but following further growth, development moves plants into a third phase in which these similarities are lost. These findings therefore highlight the importance, in all studies involving correlative phenomena, of taking into account the possible existence of regulatory changes associated with increasing structural complexity during growth.

## Supplementary material

Supplementary data can be found at *JXB* online.

Supplementary Table S1. For Experiment 1, the increase in the distal response zone in number of newly emerged leaves and stem length of the main stem.

Supplementary Table S2. For Experiment 2, the increase in branching characteristics of the four basal branches over a 14-day period following treatment application in the control, girdled, and NPA treatments.

## Funding

This work was supported in part by the MeriNet programme, New Zealand Foundation for Research, Science and Technology, grant numbers C10X0404 and C10X0816, and AgResearch Limited.

## Supplementary Material

Supplementary Data
