# Supplementary material for: Regulation of correlative inhibition of axillary bud outgrowth by basal branches varies with growth stage in Trifolium repens
Source: J Exp Bot. 2015 Apr 28;66(13):3803–13. doi: 10.1093/jxb/erv184 (PMC4473983; doi:10.1093/jxb/erv184)
Supplement: Supplementary Data [file supp_66_13_3803__index.html]

Regulation of correlative inhibition of axillary bud outgrowth by basal branches varies with growth stage in Trifolium repens — Regulation of correlative inhibition of axillary bud outgrowth by basal branches varies with growth stage in Trifolium repens — Supplementary Data 

# Regulation of correlative inhibition of axillary bud outgrowth by basal branches varies with growth stage in *Trifolium repens*

## Supplementary Data

Data files

**Files in this Data Supplement:**

- Supplementary Data - Supplementary Data
