# Supplementary material for: Regulation of correlative inhibition of axillary bud outgrowth by basal branches varies with growth stage in Trifolium repens
Source: J Exp Bot. 2015 Apr 28;66(13):3803–13. doi: 10.1093/jxb/erv184 (PMC4473983; doi:10.1093/jxb/erv184)
Supplement: Supplementary Data [file supp_erv184_jexbot142984_file001.pdf]

Supplementary material

Regulation of correlative inhibition of axillary bud outgrowth by basal branches varies with growth stage in *Trifolium repens* L.

RG THOMAS and MJM HAY

Table S1. For Experiment 1, the increase within the distal response zone in number of newly emerged leaves on (leaf no.) and stem length (mm) of the main stem. Values are given for small (S), medium (M) and large (L) plants during the 7-day period following treatment application. Basal branches either remained untreated (control) or were girdled or disbudded; n = 8. Within rows, means without a common superscript differ (P<0.05).

		Treatments		
		**Control**	**Girdled**	**Disbudded**
Leaf No.	S	1.98[a]	2.01[a]	2.01[a]
	M	1.85[a]	2.03[b]	2.01[b]
	L	1.98[a]	1.93[a]	2.00[a]
Length (mm)	S	89.3[a]	92.6[a]	96.6[a]
	M	57.4[a]	63.0[a]	70.1[b]
	L	64.6[b]	52.6[a]	74.8[c]

Table S2. For Experiment 2, the increase on the four basal branches in total number and length of secondary (2°) branches and total number of leaves on them over the 14-day period following treatment application and the number of tertiary (3°) branches forming on the secondary branches in the control, girdled and NPA treatments; n = 6. Within rows, means without a common superscript differ ($P<0.05$).

	Treatment		
	**Control**	**Girdled**	**NPA**
**2° branches**			
Number	14.4^{a}	12.4^{a}	14.7^{a}
Length	1117^{b}	848^{a}	1007^{ab}
No. of leaves	50.9^{a}	46.5^{a}	50.2^{a}
**3° branches**			
Number	4.4^{a}	3.7^{a}	4.8^{a}
